# Interdisciplinary Management of Maxillary Canine Buccal Ectopia Associated with Peg Shaped Lateral Incisor

**DOI:** 10.1155/2016/3045865

**Published:** 2016-09-20

**Authors:** Karuna Singh Sawhny, Asheesh Sawhny

**Affiliations:** ^1^Department of Orthodontics and Dentofacial Orthopedics, Rama Dental College, Hospital and Research Centre, Kanpur, Uttar Pradesh, India; ^2^Department of Conservative and Endodontic Dentistry, Rama Dental College, Hospital and Research Centre, Kanpur, Uttar Pradesh, India

## Abstract

Aligning a displaced maxillary canine into the dental arch is one of the most complicated problems in orthodontics. In cases of extremely high displacement, the tooth is frequently removed surgically. Because of the upper canines' significance to dental esthetics and functional occlusion, such a decision is a very serious one. The purpose of this report is to illustrate an interdisciplinary approach involving both orthodontic management and conservative tooth restoration. The case was treated through an orthodontic nonextraction fixed appliance mechanotherapy for successful alignment of buccally ectopic upper left canine followed by a conservative direct composite tooth buildup of peg lateral incisor associated with the upper left ectopic canine in a 16-year-old adolescent North Indian female. Posttreatment records demonstrated good alignment of the displaced tooth and restoration of normal anatomy of the peg shaped lateral incisor.

## 1. Introduction

An ectopic tooth is defined as a tooth that is following an abnormal eruption path whereas an impacted tooth is a tooth that is unable to erupt without assistance and is usually associated with an ectopic path of eruption. Since the permanent canines are the foundation of an esthetic smile and functional occlusion, their proper alignment into the arch becomes a priority for the orthodontist. The maxillary canine is present superior to the deciduous canine, angulated medially, with its crown lying distal and buccal to the lateral incisor. The canine follows a mesial path until the crown reaches the distal aspect of the lateral incisor root. The erupting canine is gradually uprighted to a more vertical position and is guided by the lateral incisor root, until it is fully erupted [1].

Maxillary canine ectopia occurs as the result of divergence from the normal path of eruption of the tooth. Primary etiological causes include disturbances in tooth eruption sequence, trauma, retention of primary canine, premature root closure, rotation of tooth buds, localized pathological lesions (cysts, odontomas) [2], long developmental path [1], more difficult and tortuous path of eruption [3], and a genetic component with recurring occurrence in some families [4] and in association with a crowded dentition [5]. But sometimes there is buccal displacement with no crowding. The cause is genetic, and the condition has been called “primary tooth germ displacement,” meaning development of the tooth bud in the aberrant position or orientation, because of an abnormal genetic pattern [6]. Such cases are usually associated with reduced mesiodistal width of the lateral incisor and increased prevalence of anomalous lateral incisor [7, 8]. The retention of the deciduous canine also aids in the deflection of the maxillary canine buccally. Rohrer [9] reported the incidence of permanent canine impaction to be 20 times higher in the maxilla than in the mandible. Hitchen [10] and Rayne [11] found that palatal impaction accounts for 85% and labial impaction 15%. The prevalence rate of buccally displaced canine is 3.06 per cent with a male-to-female ratio of 1 : 1 [12]. Shafer et al. [13] enumerated the sequel for ectopic eruption as labial or lingual malpositioning of the ectopically erupted tooth, migration of the neighbouring teeth and loss of arch length, internal resorption, dentigerous cyst formation, infection particularly with partial eruption, referred pain, and external root resorption of the impacted tooth, as well as the neighbouring teeth [14]. Following is a case of a 16-year-old female, who presented with a buccally displaced left maxillary canine which was successfully brought into the arch by orthodontic fixed appliance mechanotherapy along with direct composite buildup of the peg shaped lateral incisor associated with it.

## 2. Case History

A 16-year-old adolescent North Indian female presented with the chief complaint of the irregular teeth in relation to upper and lower arches.

## 3. Clinical Examination

**Figure 1:**
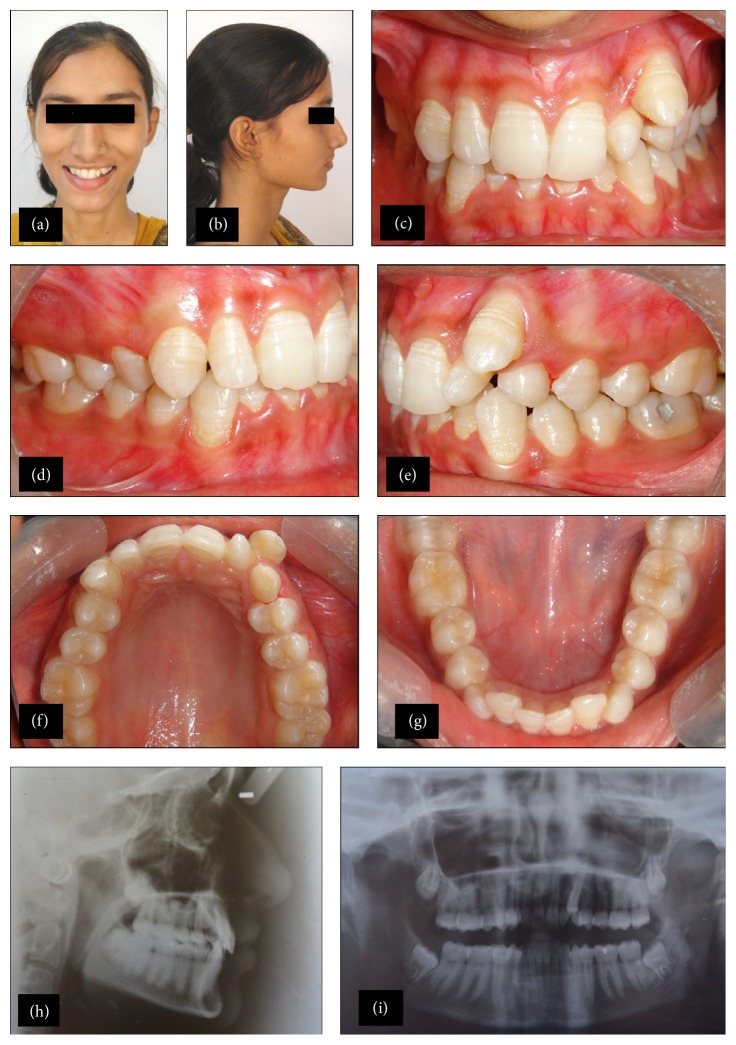
(a) Pretreatment frontal photograph. (b) Pretreatment profile photograph. (c) Pretreatment intraoral frontal view. (d) Pretreatment intraoral right lateral view. (e) Pretreatment intraoral left lateral view. (f) Pretreatment intraoral occlusal view of maxillary arch. (g) Pretreatment intraoral occlusal view of mandibular arch. (h) Pretreatment lateral cephalogram radiograph. (i) Pretreatment panoramic radiograph.

Extraoral examination showed no gross asymmetry, mesoprosopic facial form and straight profile, competent lips, and complex and nonconsonant smile with 1 mm of gingival display (Figures 1(a) and 1(b)). Intraoral examination (Figures 1(c), 1(d), 1(e), 1(f), and 1(g)) showed presence of permanent dentition till second molars, ectopically erupted 23, overretained deciduous canine (63), peg shaped lateral incisor (22), and mesiopalatally rotated 13 and mesiolabially rotated 22. Many teeth showed hypocalcification. Occlusal relationship showed Class I molar and canine relationship, 2 mm overjet and 7 mm (80%) overbite. Lower arch showed mild crowding. Maxillary dental midline coincided with the mandibular dental midline.

## 4. Radiographic Examination

The panoramic radiograph (Figure 1(h)) showed no pathologies. The maxillary and mandibular third molars were erupting and overretained 63. The lateral cephalometric analysis (Figure 1(i), Table 1) revealed that the patient had a skeletal Class I relationship with a normal growth pattern, retroclined maxillary and mandibular incisors, and retrusive upper lip.

The case was diagnosed with Class I skeletal bases: Angles Class I malocclusion with mild crowding in upper and lower anterior teeth, buccally ectopic 23, peg shaped 22, retained 63, mesiopalatally rotated 13 and mesiolabially rotated 22, retroclined upper and lower anterior teeth, decreased overjet and increased overbite, and retrusive upper lip. Clinical findings and radiographic examination indicated that the resulting buccal displacement was due to retained upper left deciduous canine and associated peg shaped permanent lateral incisor.

## 5. Treatment Objectives

The treatment objectives were to relieve the crowding in both arches, to align the buccally displaced right maxillary canine into the arch, to direct cosmetic composite buildup of the left maxillary peg shaped lateral incisor restoring it to normal anatomy, to obtain a normal overjet and overbite and pleasing smile, and to improve incisor inclination and improve facial esthetics.

## 6. Treatment Plan and Sequence

On the basis of clinical examination and diagnostic records, a treatment plan to correct the malocclusion through nonextraction fixed appliance mechanotherapy was selected. The rationale was to gain space for mild crowding by extraction of retained deciduous canine (63) and proclination of upper and lower incisors, to avoid compromising the patient's profile and to shorten the treatment time.

**Figure 2:**
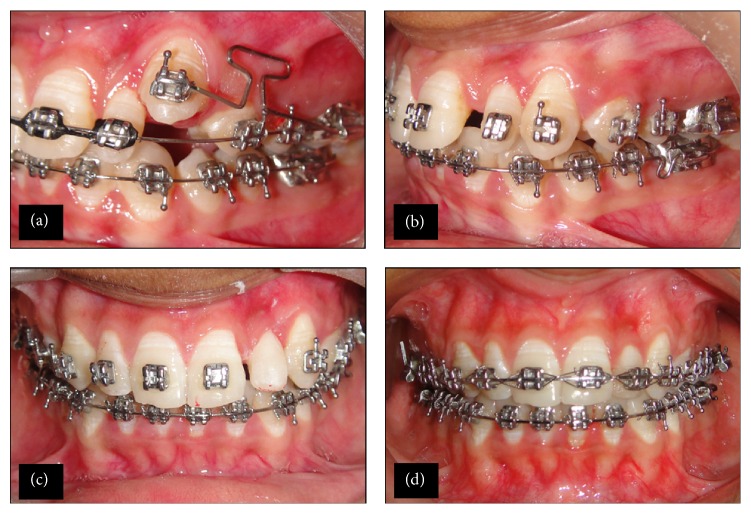
(a) Auxiliary T-loop (0.0175 × 0.025′′ TMA wire). (b) Alignment of canine in the arch. (c) Direct composite build up of peg shaped lateral incisor (22). (d) Complete levelling and alignment of arches.

Overretained deciduous canine (63) was extracted. Full fixed 0.022 inch MBT 0.0175 × 0.025′′ TMA wire brackets were bonded on both arches except 23. After initial levelling and alignment with 0.014, 0.016, and 0.018 nickel-titanium HANT archwires, both the arches were stabilized with coordinated 0.018′′ stainless steel wires. 23 was bonded. An auxiliary segmented T-loop made from 0.022 inch MBT 0.0175 × 0.025′′ TMA wire (Figure 2(a)) from 26 was attached to 23 and its mesial arm was activated in distal, occlusal, and palatal direction to bring the buccally displaced right maxillary canine in the arch. Final alignment of the 23 was done using a piggy back 0.014′′ NiTi archwire. Slight space was opened between 21 and 22 with NiTi open coil spring (Figure 2(b)). After 10 months of the treatment, the bracket in relation to 22 was deboned and referred to restorative dentist for direct composite build of peg shaped 22. A light cure adhesive system (3M™ ESPE Scotch Bond™ Universal Adhesive, Neuss, Germany) was applied. The buildup was done by incremental placement of restorative composite (3M ESPE Filtek™ Z 250 XT Nano Hybrid Universal Restorative, Neuss, Germany, A2 shade), followed by contouring and light curing. Finally, the restoration was finished and polished (Figure 2(c)).

22 was rebonded, levelled, and aligned and slight space mesial to 22 was closed (Figure 2(c)). Coordinated 0.019 × 0.025′′ SS was placed with Class II elastic on left side. Final finishing wires 0.14′′ NiTi were placed. Total treatment time was 15 months, followed by bonding of a maxillary (to keep the buccally displaced right maxillary canine in optimal position) and mandibular fixed spiral wire retainer.

## 7. Treatment Results

**Figure 3:**
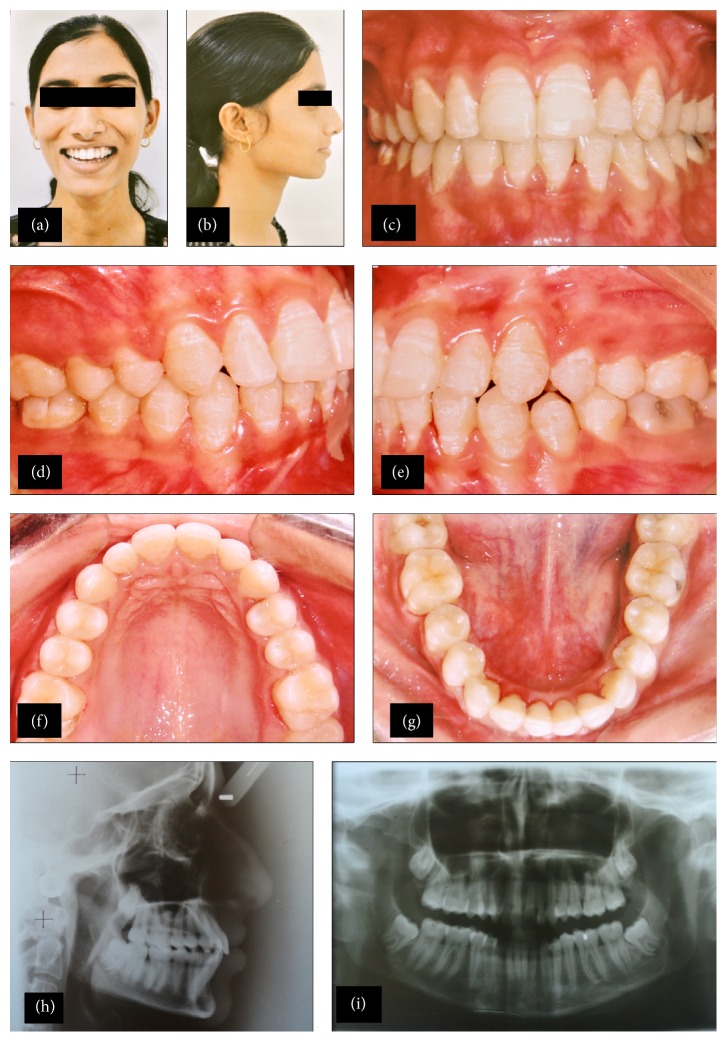
(a) Posttreatment frontal photograph (smiling). (b) Posttreatment profile photograph. (c) Posttreatment intraoral frontal photograph. (d) Posttreatment intraoral right lateral photograph. (e) Posttreatment intraoral left lateral photograph. (f) Posttreatment intraoral maxillary occlusal photograph. (g) Posttreatment intraoral mandibular occlusal photograph. (h) Posttreatment lateral cephalogram. (i) Posttreatment panoramic radiograph.

Posttreatment records revealed that treatment objectives were achieved. Facial photographs showed an improved profile and smile (Figures 3(a) and 3(b)). Class I canine was established with canine-protected occlusion. Dental midlines were aligned with the facial midline, with ideal overbite and overjet (Figures 3(c), 3(d), 3(e), 3(f), and 3(g)). Posttreatment panoramic radiograph showed acceptable root parallelism, with no signs of bone or root resorption (Figures 3(h) and 3(i)).

Posttreatment lateral cephalometric analysis (Table 1) and superimposition revealed a Class I skeletal pattern, improved inclinations of maxillary and mandibular incisors, ideal overbite and overjet, and improvement in upper lip protrusion.

## 8. Discussion

The upper canine despite being the tooth of most frequent eruption anomalies after third molars was considered as one of the most important teeth of the dental arch. For a good treatment prognosis, the proper evaluation of canine position must be done through clinical and radiographic evaluation and treatment alternatives analyzed according to the particularities of each case after careful evaluation of the orthodontist and professionals of distinct areas. Various methods have been suggested for the traction and alignment of impacted canines, among which are the orthodontic removable or fixed appliances and the use of anchorage in the same arch or opposite arch. However, a fixed orthodontic appliance provides greater control and effectiveness of the force applied, and in most of the cases there is a need to correct some other type of associated malocclusion and to open and keep the space to apply traction on the tooth, using specific accessories as loops. With regard to the force system for traction of impacted or ectopic canines, one must be careful with the direction of the applied force, because this should not direct traction to the roots of neighbouring teeth, not to cause trauma and external root resorptions. It is recommended, initially, to gain space in dental arches before traction and also the use of force of low intensity (no more than 60 g) and the employment of sufficiently rigid archwires to prevent deflection which may undermine movement control [15].

Direct composite bonding is a good treatment option for peg shaped laterals as it is conservative and can be placed directly onto the tooth. It also preserves sound tooth structure, is long lasting, can be repaired easily, and is cost effective [16, 17].

The present case uses cantilever system that provides the proper control for canine movement, associated with a smaller load on anchorage units. It is constructed with titanium-molybdenum wire (TMA) of 0.017 × 0.025 inches. The advantage found in this method consists in the ability to work with a defined force system [18]. To avoid relapse in the future, bonded retainers are the retainer of choice. Becker et al. evaluated posttreatment alignment cases whose treatment was completed. This would support fixed retention in this case and in many cases where the relapse potential is increased [19].

## 9. Conclusion

The successful treatment of a patient with an ectopic tooth can be a challenging task for an orthodontist. This case report has demonstrated careful planning in alignment of the buccally displaced canine with mild crowding and peg shaped lateral incisor by nonextraction fixed orthodontic mechanics to deliver light, controlled force, with good results. Thus, the planned orthodontic treatment resulted in correction of occlusion, harmony of smile, periodontal health, and stability after treatment.

## Figures and Tables

**Table 1:** SNA: sella nasion to point A; SNB: sella nasion to point B; ANB: difference of SNA and SNB; Wits: perpendicular from point A and point B occlusal plane; FMA: FH plane to mandibular plane; *y*-axis: FH plane to sella gnathion plane; U1-NA: upper incisor inclination to nasion-point A plane; L1-NB: lower incisor inclination to nasion-point B plane.

Cephalometric variables	Pretreatment values	Posttreatment values
(1) SNA	81°	80°
(2) SNB	79°	77°
(3) ANB	2°	3°
(4) Wits	0 mm	0 mm
(5) FMA	25°	26°
(6) *y*-axis	61°	59
(7) U1-NA (°)	19°	25°
(8) U1-NA (mm)	3 mm	+5 mm
(9) L1-NB (°)	17°	28°
(10) L1-NB (mm)	1 mm	6 mm
(11) Upper lip–E-line (mm)	−4.5 mm	−2 mm
(12) Lower lip–E-line (mm)	+1 mm	+2 mm
